# 5-Amino-1*H*-1,2,4-triazol-4-ium hydrogen oxalate

**DOI:** 10.1107/S1600536813019363

**Published:** 2013-07-20

**Authors:** Manel Essid, Houda Marouani, Salem S. Al-Deyab, Mohamed Rzaigui

**Affiliations:** a Laboratoire de Chimie des Matériaux, Faculté des Sciences de Bizerte, 7021 Zarzouna Bizerte, Tunisia; b Chemistry Department, Faculty of Science, King Saud University, PO Box 2455, Riyadh 11451, Saudi Arabia

## Abstract

In the title salt, C_2_H_5_N_4_^+^·C_2_HO_4_^−^, the hydrogen oxalate anions form corrugated chains parallel to the *c* axis, linked by inter­molecular O—H⋯O hydrogen bonds. The 5-amino-1*H*-1,2,4-triazol-4-ium cations are connected into centrosymmetric clusters *via* weak C—H⋯N hydrogen bonds forming nine-membered rings with an *R*_3_^3^(9) motif. These clusters are inter­connected *via* anions through N—H⋯O hydrogen bonds, building a three-dimensional network.

## Related literature

For the properties of triazoles, see: Li *et al.* (2004 ▶); Mernari *et al.* (1998 ▶); Bentiss *et al.* (1999 ▶). For graph-set notation of hydrogen bonding, see: Bernstein *et al.* (1995 ▶). For related structures, see: Matulková *et al.* (2007 ▶, 2008 ▶).
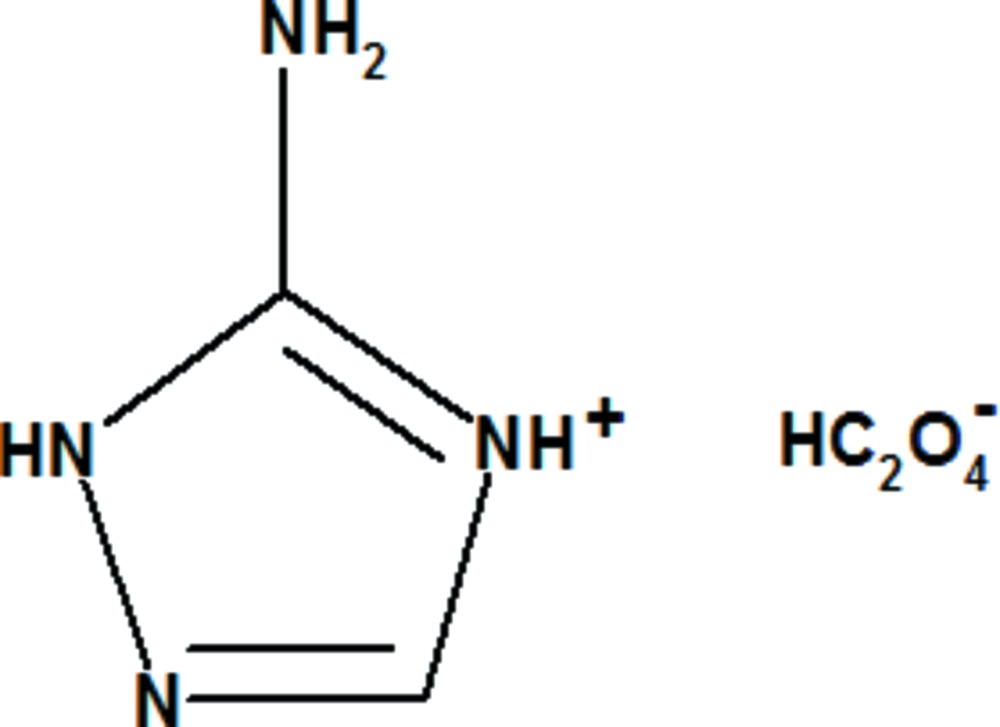


## Experimental

### 

#### Crystal data

C_2_H_5_N_4_^+^·C_2_HO_4_^−^
*M* *_r_* = 174.13
Trigonal, 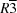

*a* = 23.093 (4) Å
*c* = 6.603 (3) Å
*V* = 3049.3 (16) Å^3^
*Z* = 18
Ag *K*α radiation
λ = 0.56080 Å
μ = 0.09 mm^−1^
*T* = 293 K
0.35 × 0.3 × 0.25 mm

#### Data collection

Enraf–Nonius CAD-4 diffractometer
3909 measured reflections
3313 independent reflections
1929 reflections with *I* > 2σ(*I*)
*R*_int_ = 0.023
2 standard reflections every 120 min intensity decay: 1%

#### Refinement

*R*[*F* ^2^ > 2σ(*F* ^2^)] = 0.060
*wR*(*F* ^2^) = 0.194
*S* = 1.04
3313 reflections
121 parameters
H atoms treated by a mixture of independent and constrained refinement
Δρ_max_ = 0.50 e Å^−3^
Δρ_min_ = −0.42 e Å^−3^

### 

Data collection: *CAD-4 EXPRESS* (Enraf–Nonius, 1994 ▶); cell refinement: *CAD-4 EXPRESS*; data reduction: *XCAD4* (Harms & Wocadlo, 1995 ▶); program(s) used to solve structure: *SHELXS86* (Sheldrick, 2008 ▶); program(s) used to refine structure: *SHELXL97* (Sheldrick, 2008 ▶); molecular graphics: *ORTEP-3 for Windows* (Farrugia, 2012 ▶); software used to prepare material for publication: *WinGX* (Farrugia, 2012 ▶).

## Supplementary Material

Crystal structure: contains datablock(s) I, global. DOI: 10.1107/S1600536813019363/pv2640sup1.cif

Structure factors: contains datablock(s) I. DOI: 10.1107/S1600536813019363/pv2640Isup2.hkl

Supplementary material file. DOI: 10.1107/S1600536813019363/pv2640Isup3.cml

Additional supplementary materials:  crystallographic information; 3D view; checkCIF report

## Figures and Tables

**Table 1:** Hydrogen-bond geometry (Å, °)

*D*—H⋯*A*	*D*—H	H⋯*A*	*D*⋯*A*	*D*—H⋯*A*
O1—H1⋯O3^i^	0.87 (3)	1.72 (3)	2.5845 (17)	174 (3)
N1—H2⋯O1^ii^	0.86 (3)	2.29 (3)	3.087 (2)	155 (2)
N1—H3⋯O4^iii^	0.88 (3)	2.06 (3)	2.925 (2)	171 (3)
N2—H4⋯O4^iv^	0.86	2.09	2.892 (2)	154
N2—H4⋯O2^iv^	0.86	2.28	2.878 (2)	127
N3—H5⋯O3^iii^	0.86	1.94	2.7652 (18)	161
C4—H6⋯N4^v^	0.93	2.41	3.313 (3)	165

Symmetry codes: (i) 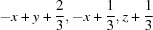; (ii) 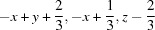; (iii) 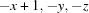; (iv) 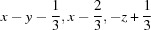; (v) 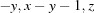.

**Fig. 1.**
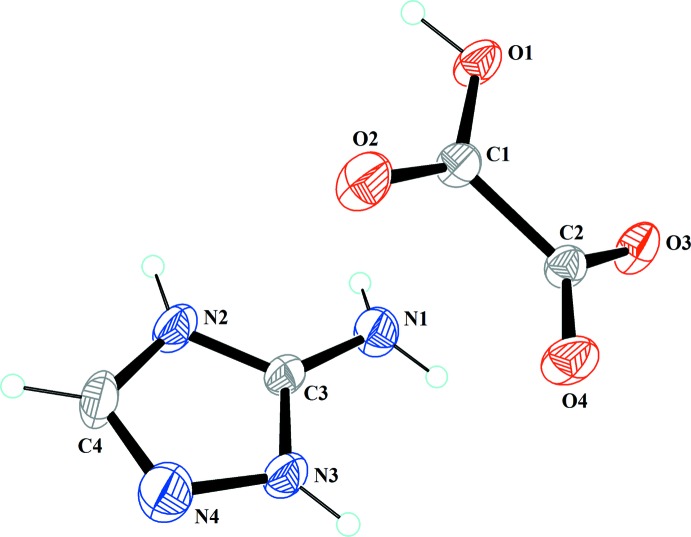
An ORTEP view of the title salt with the atom-labelling scheme. Displacement ellipsoids are drawn at the 30% probability level. H atoms are represented as small spheres of arbitrary radii.

**Fig. 2.**
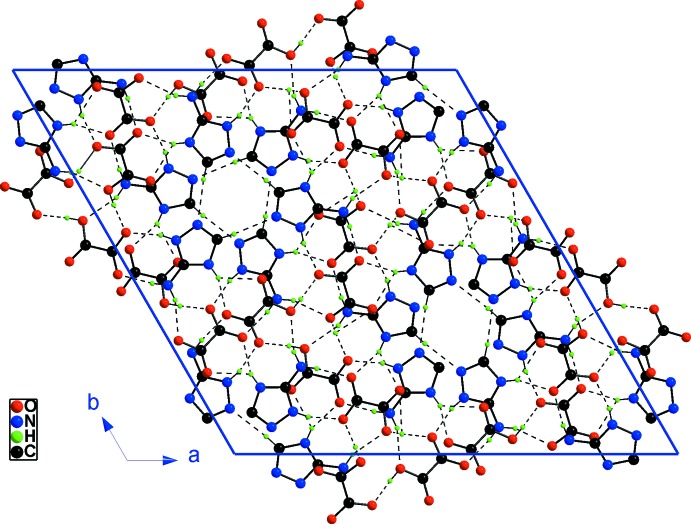
A view of the hydrogen bonds (dotted lines) in the crystal structure of the title salt. H atoms non-participating in hydrogen- bonding were omitted for clarity.

## supplementary crystallographic information

### Comment

Triazole derivatives are used in the synthesis of antibiotics, fungicides, herbicides, plant growth hormone regulators (Li *et al.*, 2004), and potentially good corrosion inhibitors (Mernari *et al.*, 1998; Bentiss *et al.*, 1999). Materials based on triazole compounds with dicarboxylic acids (4-amino-1,2,4-triazol-1-ium oxalate, adducts of 4-amino-1,2,4-triazole with succinic acid and adipic acid and 3-amino-1,2,4-triazolinium hydrogen *L*-tartrate) were also prepared and characterized as promising compounds in the field of non linear optics (Matulková *et al.*, 2008; Matulková *et al.*, 2007).

The asymmetric unit of the title salt (Fig. 1) contains a 5-amino-1,2,4-triazol-4-ium cation and an oxalate anion. The cation is monoprotonated at atom N2 and oxalic acid is mono-deprotonated. Geometrical parameters of the cation are found to be in agreement with those of other similar structures of 3-amino-1,2,4-triazolinium(1+) hydrogen *L*-tartrate (Matulková *et al.*, 2007).

The crystal structure is based on a three dimensional network of hydrogen oxalic acid anions interconnected by O—H···O hydrogen bonds with lengths of 2.585 Å.

Planar 5-amino-1,2,4-triazolinium cations are located in the cavities of the hydrogen oxalic acid network and connected with anions *via* linear and bifurcated N—H···O hydrogen bonds. The donor-acceptor distances in these hydrogen bonds attain values from 2.765 to 3.087 Å (Tab. 1 and Fig. 2).

The oxalate ion is maintained by moderate hydrogen bonds that link the oxygen atoms of oxalate ion and the hydrogen of the other oxalate into corrugated chains parallel to the *c* axis. In addition, there are weak C—H···N hydrogen bonds in the crystal structure between 5-amino-1,2,4-triazilium cations forming an *R*_3_^3^(9) motif (Fig. 2) (Bernstein *et al.*, 1995). These cations are interconnected *via* anions through N—H···O hydrogen bonds, building a three dimensional network.

### Experimental

An aqueous solution of H_2_C_2_O_4_ (2 mmol in 10 ml water) was added to an aqueous solution of 5-amino-1*H*-1,2,4-triazole (2 mmol in 10 ml of water). The obtained solution was stirred at 333 K for 30 min and then left to stand at room temperature. Colorless single crystals of the title compound were obtained after some days.

### Refinement

The hydrogen atoms bonded to O1 and N1 were located from a difference map and were allowed to refine. The rest of the H atoms were treated as riding, with C—H = 0.93 Å and N—H = 0.86 Å and with *U*_iso_(H) = 1.2*U*_eq_(C or N).

### Crystal data

**Table:**

C_2_H_5_N_4_^+^·C_2_HO_4_^−^	*D*_x_ = 1.707 Mg m^−^^3^
*M**_r_* = 174.13	Ag *K*α radiation, λ = 0.56080 Å
Trigonal, *R*3	Cell parameters from 25 reflections
Hall symbol: -R 3	θ = 9–11°
*a* = 23.093 (4) Å	µ = 0.09 mm^−^^1^
*c* = 6.603 (3) Å	*T* = 293 K
*V* = 3049.3 (16) Å^3^	Prism, colorless
*Z* = 18	0.35 × 0.3 × 0.25 mm
*F*(000) = 1620	

### Data collection

**Table:**

Enraf–Nonius CAD-4 diffractometer	*R*_int_ = 0.023
Radiation source: fine-focus sealed tube	θ_max_ = 28.0°, θ_min_ = 2.4°
Graphite monochromator	*h* = −1→33
non–profiled ω scans	*k* = −1→33
3909 measured reflections	*l* = −11→11
3313 independent reflections	2 standard reflections every 120 min
1929 reflections with *I* > 2σ(*I*)	intensity decay: 1%

### Refinement

**Table:**

Refinement on *F*^2^	Primary atom site location: structure-invariant direct methods
Least-squares matrix: full	Secondary atom site location: difference Fourier map
*R*[*F*^2^ > 2σ(*F*^2^)] = 0.060	Hydrogen site location: inferred from neighbouring sites
*wR*(*F*^2^) = 0.194	H atoms treated by a mixture of independent and constrained refinement
*S* = 1.04	*w* = 1/[σ^2^(*F*_o_^2^) + (0.0979*P*)^2^ + 1.6007*P*] where *P* = (*F*_o_^2^ + 2*F*_c_^2^)/3
3313 reflections	(Δ/σ)_max_ < 0.001
121 parameters	Δρ_max_ = 0.50 e Å^−^^3^
0 restraints	Δρ_min_ = −0.42 e Å^−^^3^

### Special details

**Table:**

Geometry. All e.s.d.'s (except the e.s.d. in the dihedral angle between two l.s. planes) are estimated using the full covariance matrix. The cell e.s.d.'s are taken into account individually in the estimation of e.s.d.'s in distances, angles and torsion angles; correlations between e.s.d.'s in cell parameters are only used when they are defined by crystal symmetry. An approximate (isotropic) treatment of cell e.s.d.'s is used for estimating e.s.d.'s involving l.s. planes.
Refinement. Refinement of *F*^2^ against ALL reflections. The weighted *R*-factor *wR* and goodness of fit *S* are based on *F*^2^, conventional *R*-factors *R* are based on *F*, with *F* set to zero for negative *F*^2^. The threshold expression of *F*^2^ > σ(*F*^2^) is used only for calculating *R*-factors(gt) *etc*. and is not relevant to the choice of reflections for refinement. *R*-factors based on *F*^2^ are statistically about twice as large as those based on *F*, and *R*- factors based on ALL data will be even larger.

### Fractional atomic coordinates and isotropic or equivalent isotropic displacement parameters (Å^2^)

**Table:**

	*x*	*y*	*z*	*U*_iso_*/*U*_eq_	
O1	0.35441 (6)	−0.03841 (6)	0.4263 (2)	0.0279 (3)	
O4	0.50310 (6)	−0.02945 (6)	0.2759 (2)	0.0304 (3)	
O3	0.46709 (6)	0.04345 (6)	0.2350 (2)	0.0268 (3)	
O2	0.37748 (7)	−0.12148 (6)	0.4035 (3)	0.0384 (4)	
N3	0.40979 (7)	−0.14645 (7)	−0.1112 (2)	0.0282 (3)	
H5	0.4515	−0.1183	−0.1295	0.034*	
N1	0.36951 (8)	−0.06980 (8)	−0.0972 (3)	0.0310 (3)	
N2	0.30499 (7)	−0.18848 (8)	−0.0659 (3)	0.0308 (3)	
H4	0.2661	−0.1927	−0.0501	0.037*	
C3	0.36142 (7)	−0.13134 (8)	−0.0947 (2)	0.0228 (3)	
C2	0.46067 (7)	−0.01195 (7)	0.2875 (2)	0.0205 (3)	
C4	0.31868 (9)	−0.23999 (9)	−0.0656 (3)	0.0338 (4)	
H6	0.2877	−0.2851	−0.0480	0.041*	
C1	0.39246 (8)	−0.06374 (7)	0.3785 (2)	0.0217 (3)	
N4	0.38176 (9)	−0.21484 (9)	−0.0939 (3)	0.0429 (4)	
H1	0.3155 (15)	−0.0699 (15)	0.467 (4)	0.054 (8)*	
H3	0.4074 (14)	−0.0368 (14)	−0.142 (4)	0.049 (7)*	
H2	0.3342 (14)	−0.0667 (12)	−0.113 (4)	0.039 (6)*	

### Atomic displacement parameters (Å^2^)

**Table:**

	*U*^11^	*U*^22^	*U*^33^	*U*^12^	*U*^13^	*U*^23^
O1	0.0205 (5)	0.0209 (5)	0.0446 (7)	0.0121 (4)	0.0104 (5)	0.0068 (5)
O4	0.0229 (5)	0.0301 (6)	0.0442 (7)	0.0179 (5)	0.0058 (5)	0.0057 (5)
O3	0.0183 (5)	0.0208 (5)	0.0405 (7)	0.0091 (4)	0.0014 (5)	0.0076 (5)
O2	0.0334 (7)	0.0216 (6)	0.0648 (10)	0.0172 (5)	0.0158 (6)	0.0122 (6)
N3	0.0185 (6)	0.0235 (6)	0.0423 (8)	0.0103 (5)	0.0040 (5)	0.0025 (6)
N1	0.0276 (7)	0.0244 (7)	0.0437 (9)	0.0149 (6)	−0.0001 (6)	0.0016 (6)
N2	0.0159 (5)	0.0274 (7)	0.0448 (9)	0.0076 (5)	0.0030 (5)	0.0023 (6)
C3	0.0170 (6)	0.0235 (7)	0.0270 (7)	0.0093 (5)	0.0004 (5)	−0.0004 (5)
C2	0.0177 (6)	0.0206 (6)	0.0237 (7)	0.0101 (5)	0.0000 (5)	0.0008 (5)
C4	0.0219 (7)	0.0183 (7)	0.0541 (12)	0.0047 (6)	0.0015 (7)	0.0026 (7)
C1	0.0203 (6)	0.0195 (6)	0.0279 (7)	0.0120 (5)	0.0022 (5)	0.0037 (5)
N4	0.0336 (9)	0.0314 (8)	0.0665 (12)	0.0184 (7)	0.0035 (8)	0.0027 (8)

### Geometric parameters (Å, º)

**Table:**

O1—C1	1.3143 (19)	N1—H3	0.88 (3)
O1—H1	0.87 (3)	N1—H2	0.86 (3)
O4—C2	1.2351 (19)	N2—C3	1.325 (2)
O3—C2	1.2607 (18)	N2—C4	1.375 (2)
O2—C1	1.2099 (19)	N2—H4	0.8600
N3—C3	1.331 (2)	C2—C1	1.546 (2)
N3—N4	1.380 (2)	C4—N4	1.284 (3)
N3—H5	0.8600	C4—H6	0.9300
N1—C3	1.338 (2)		
			
C1—O1—H1	110.1 (19)	N3—C3—N1	126.00 (15)
C3—N3—N4	108.56 (14)	O4—C2—O3	127.23 (15)
C3—N3—H5	125.7	O4—C2—C1	116.06 (13)
N4—N3—H5	125.7	O3—C2—C1	116.71 (13)
C3—N1—H3	118.4 (19)	N4—C4—N2	107.94 (15)
C3—N1—H2	117.1 (17)	N4—C4—H6	126.0
H3—N1—H2	118 (2)	N2—C4—H6	126.0
C3—N2—C4	108.94 (14)	O2—C1—O1	124.85 (15)
C3—N2—H4	125.5	O2—C1—C2	121.67 (14)
C4—N2—H4	125.5	O1—C1—C2	113.47 (12)
N2—C3—N3	106.69 (14)	C4—N4—N3	107.86 (15)
N2—C3—N1	127.24 (15)		
			
C4—N2—C3—N3	−0.3 (2)	O3—C2—C1—O2	168.06 (17)
C4—N2—C3—N1	−177.35 (19)	O4—C2—C1—O1	166.66 (15)
N4—N3—C3—N2	0.6 (2)	O3—C2—C1—O1	−12.8 (2)
N4—N3—C3—N1	177.71 (18)	N2—C4—N4—N3	0.5 (2)
C3—N2—C4—N4	−0.1 (2)	C3—N3—N4—C4	−0.7 (2)
O4—C2—C1—O2	−12.5 (2)		

### Hydrogen-bond geometry (Å, º)

**Table:**

*D*—H···*A*	*D*—H	H···*A*	*D*···*A*	*D*—H···*A*
O1—H1···O3^i^	0.87 (3)	1.72 (3)	2.5845 (17)	174 (3)
N1—H2···O1^ii^	0.86 (3)	2.29 (3)	3.087 (2)	155 (2)
N1—H3···O4^iii^	0.88 (3)	2.06 (3)	2.925 (2)	171 (3)
N2—H4···O4^iv^	0.86	2.09	2.892 (2)	154
N2—H4···O2^iv^	0.86	2.28	2.878 (2)	127
N3—H5···O3^iii^	0.86	1.94	2.7652 (18)	161
C4—H6···N4^v^	0.93	2.41	3.313 (3)	165

Symmetry codes: (i) −*x*+*y*+2/3, −*x*+1/3, *z*+1/3; (ii) −*x*+*y*+2/3, −*x*+1/3, *z*−2/3; (iii) −*x*+1, −*y*, −*z*; (iv) *x*−*y*−1/3, *x*−2/3, −*z*+1/3; (v) −*y*, *x*−*y*−1, *z*.
